# Robots and AI as Legal Subjects? Disentangling the Ontological and Functional Perspective

**DOI:** 10.3389/frobt.2022.842213

**Published:** 2022-04-05

**Authors:** Andrea Bertolini, Francesca Episcopo

**Affiliations:** ^1^ Scuola Superiore Sant’Anna, Dirpolis Institute, Pisa, Italy; ^2^ Università di Pisa, Department of Private Law and Scuola Superiore Sant’Anna, Dirpolis Institute, Pisa, Italy

**Keywords:** legal subjects, personhood, agency, responsibility, autonomy, liability, electronic personhood, risk-management

## Abstract

Robotics and AI-based applications (RAI) are often said to be so technologically advanced that they should be held responsible for their actions, instead of the human who designs or operates them. The paper aims to prove that this thesis (“the exceptionalist claim”)—as it stands—is both theoretically incorrect and practically inadequate. Indeed, the paper argues that such claim is based on a series of misunderstanding over the very notion and functions of “legal responsibility”, which it then seeks to clarify by developing and interdisciplinary conceptual taxonomy. In doing so, it aims to set the premises for a more constructive debate over the feasibility of granting legal standing to robotic application. After a short Introduction setting the stage of the debate, the paper addresses the ontological claim, distinguishing the philosophical from the legal debate on the notion of i) subjectivity and ii) agency, with their respective implications. The analysis allows us to conclude that the attribution of legal subjectivity and agency are purely fictional and technical solutions to facilitate legal interactions, and is not dependent upon the intrinsic nature of the RAI. A similar structure is maintained with respect to the notion of responsibility, addressed first in a philosophical and then legal perspective, to demonstrate how the latter is often utilized to both pursue ex ante deterrence and ex post compensation. The focus on the second objective allows us to bridge the analysis towards functional (law and economics based) considerations, to discuss how even the attribution of legal personhood may be conceived as an attempt to simplify certain legal interactions and relations. Within such a framework, the discussion whether to attribute legal subjectivity to the machine needs to be kept entirely within the legal domain, and grounded on technical (legal) considerations, to be argued on a functional, bottom-up analysis of specific classes of RAI. That does not entail the attribution of animacy or the ascription of a moral status to the entity itself.

## Introduction

Whether advanced robots and AI applications (henceforth, RAI) are, should, and eventually will be considered as “subjects” rather than mere “objects” is a question that has strongly characterized the social, philosophical, and legal debate since Solum’s seminar article on “Legal Personhood for Artificial Intelligence” (Solum, 1992), and arguably even earlier (Turing, 1950; Putman, 1964; Nagel, 1974; Bunge, 1977; Taylor, 1977; Searle, 1980; Searle, 1984; McNally and Inayatullah, 1988). However, debates have significantly intensified over the last two decades, with interest in both the scientific and non-academic circles raising every time a new technology rolls out (e.g., autonomous cars being tested in real-life scenarios on our streets), or an outstanding socio-legal development occurs (e.g., the humanoid Sophia receiving Saudi Arabian citizenship)^1^ (see, e.g., Allen et al., 2000; Allen et al., 2005; Teubner, 2006; Chrisley, 2008; Coeckelbergh, 2010; Koops et al., 2010; Gunkel, 2012; Basl, 2014; Balkin, 2015a; Iannì and Monterossi, 2017; Christman, 2018; Gunkel, 2018; Nyholm, 2018; Pagallo, 2018b; Santoni de Sio and van den Hoven, 2018; Lior, 2019; Loh, 2019; Turner, 2019; Wagner, 2019; Andreotta, 2021; Basl et al., 2020; Bennett and Daly, 2020; Dignum, 2020; Gunkel, 2020; Kingwell, 2020; Osborne, 2020; Powell, 2020; Serafimova, 2020; Wheeler, 2020; De Pagter, 2021; Gabriel, 2021; Gogoshin, 2021; Gordon, 2021; Gunkel and Wales, 2021; Joshua, 2021; Kiršienė et al., 2021; Martínez and Winter 2021; Schröder, 2021; Singer, 2021).

In the policymaking arena, a recommendation from the European Parliament famously urged the European Commission to consider whether robots could be attributed an “electronic personality” (European Parliament, 2017), but the idea didn’t gain momentum and found no place in the most recent initiatives on the regulation of RAI, some of which seem to dismiss the possibility in a surprisingly sweeping fashion (European Commission, 2018; European Parliament, 2020). Yet, with social robots soon to be incorporated into our lives, a sound discussion of whether—to borrow the Editors’ own words—“robots, AI, or other socially interactive, autonomous systems have [or will ever have] some claim to moral and legal standing”^2^ becomes inescapable.

Engaging with some of the most prominent literature in the field, the paper seeks to answer the second prong of this question, i.e., whether robots, AI, or socially interactive, autonomous system have some claim to *legal* standing.

The contribution that the paper seeks to make is threefold.

First, the paper develops a specific framework to disentangle the conceptual and analytical knots, whose obfuscating presence often misleads even the most insightful analyses of the matter. The framework is based on three major distinctions, which the vast and heterogenous debate on RAI’s standing needs to acknowledge and take into consideration: i) between the legal and the moral domain, and between the respective notions of “responsible subject”; ii) between the fully fledged and the limited notions of subjectivity; iii) between the ontological/essentialist and the functionalist/consequentialist grounds of standing.

Secondly, the paper discusses some fundamental concepts which come into play in the discussion of moral and legal standing of RAI—i.e., those of agency, responsibility, and personality—, to lay the ground for a shared understanding of the debate.

Thirdly, and applying the methodological and conceptual tools described above, the paper argues that: i) at the current stage, there are no ontological reasons why RAI need to be considered legal subjects; and ii) there may nevertheless be functional reasons to do so in particular cases, when endowing them with specific rights and obligations proves the best way of fostering the individual and social interests that the law is meant to protect.

Against this backdrop, the paper is structured as follows.

In §2 we introduce some of the traditional claims for treating RAI as subjects and identify a series of conceptual and analytical problems. Moving from these considerations, we sketch the analytical framework shape distinguish the various perspectives, which we believe that a sound a coherent discussion of RAI’s standing should follow.

In §3, we put said analytical framework into practice. We first narrow down the ultimate scope of the inquiry, relating to a generalized moral and legal standing, but rather to RAI’s specific capacity to qualify as subjects legally accountable for the illegal or wrongful actions and events caused. Accordingly, we disentangle the legal from the moral dimension of standing and move on to consider when an entity may be granted a particular legal qualification and be subjected to a given legal regime, separating what we refer to as, respectively, the ontological and the functional viewpoints.

Following this line of argumentation, §4 proves that, at this stage, there are no ontological reasons to consider RAI as legal subjects. §5 then adopts the functional perspective and argues that, despite ontologically qualifying as objects and not subjects, it may nevertheless be appropriate and desirable, under certain circumstances, to grant specific technological applications with limited and narrow forms of legal personality.

In conclusion, §6 sums up the main arguments and uses them to critically discuss the European Parliament proposal of October 2020 (European Parliament, 2020), which seems to categorically exclude the possibility to treat RAI as legal subjects.

## Touring the RAI’s Subjectivity Forest: An Analytical Framework

The literature on RAI’s subjectivity is vast and varied. However, two threads seem particularly relevant.

On the one hand, it has been claimed that a robot, an intelligent artefact, or other socially interactive mechanisms, may be due to some level of social standing or respect. That they seem to have the “psychological capacities that we had previously thought were reserved for complex biological organism such as humans” (Prescott, 2017). That they are “worthy of moral value,” if not moral subjects *tout court*, and that not giving them legal standing would constitute a violation of their rights, as well as an impoverishment of our ethical stance as human beings. In these terms, the fight for RAI’s rights is frequently framed as another step in the corrective evolution of our legal systems, which has progressively expanded the legal recognition of previously discriminated humans, and is now opening towards non-human entities—animals, rivers, idols, etc.—(Gunkel, 2018; Kurki, 2019; Gellers, 2021).

At the same time, it has been claimed that some RAI are so technologically advanced, that they invite “a systemic change to laws or legal institutions in order to preserve or rebalance established values” (Calo, 2015, 553). In this sense, they should be recognized as subjects, having rights and duties of their own comparable, if not identical, to those of natural persons (Floridi and Sanders, 2004; Matthias, 2004; Stahl, 2006; Teubner, 2006; Matthias, 2008; Koops et al., 2010; Matthias, 2010; Gunkel, 2012; Floridi, 2014; Calo, 2015; Schwitzgebel and Garza, 2015; Richards and Smart, 2016; Gunkel, 2018; Nyholm, 2018; Danaher, 2020; Gunkel, 2020; Nyholm, 2020; Gunkel and Wales, 2021). In particular, it is often argued that being their actions so much outside humans’ control, we should deem them responsible for the wrong caused, instead of blaming the producer, the owner, or the user behind them (Matthias, 2004; Stahl, 2006; Matthias, 2008; Purves et al., 2015; De Jong, 2020; Gunkel, 2020).

Having a comprehensive view of the debate is particularly important, as it shows the plurality of concerns at stake in the discussion on the status of RAI in our society, whose viewpoints and analytical tools overlap and complement one another only in part. With a certain degree of approximation, there are three orthogonal strands of analysis worth identifying.

First, the current debate on the subjectivity of RAI sits at the crossroad of different disciplines: engineering, computer science, law, philosophy, sociology, to name but a few. If cross-fertilization and plurality of perspectives are to be fully welcomed, some caveats are needed to avoid negative side-effects.

Secondly, in social sciences, the debate on the subjectivity of RAI is shaped around a series of overlapping concerns and questions. In our opinion, the most important distinction to be drawn is the one between those who discuss what moral and legal entitlements RAI may and possibly should be granted as the main research question, and those who come to it indirectly, as part of an inquiry which has the focus somewhere else—in the example above, the allocation of responsibility for illegal or wrongful actions and events. The very interest and sensibility between the two viewpoints differ radically: asking the broad theoretical question of whether robots have a claim to moral and legal entitlements not only is broader in its scope and implications, but often responds to a peculiar “robot-centered” approach: despite considering both positive and negative entitlements the starting point is commonly the recognition of robot’s *rights* for the robot’s own sake, or at least for a coherent and correct explication of the moral or legal system. Conversely, those who discuss the issue of robotics’ entitlements indirectly, as a means of addressing specific problems, often have a “human-centered” standpoint: raising robots to the status of subjects is commonly presented as a way of solving what we, as humans, consider a moral or legal problem.

Thirdly, and finally, the debate on the subjectivity of RAI may be distinguished based on the grounds according to which the latter are considered as worthy (or unworthy) of raising to the status of subjects. We identified two major approaches, which we call, respectively, “ontological” or “essentialist,” and “functional’ or “consequentialist”. The first answers the question of RAI’s standing moving from the properties they display, while the other bases its answer on the consequences which derive from their legal qualification.

Taken together, these distinctions are of fundamental importance. Not only do they work as critical tools, allowing us to dissect and fully understand the state of the discussion—what claims exactly are made, for which purposes’ and upon which grounds. They also constitute essential tools for constructing a sound analytical framework, which could help us address the question of electronic personhood.

Differentiating law and morality teaches us two lessons. One is conceptual: we need to avoid the temptation to automatically translate assumptions or standards pertaining, e.g., to moral philosophy, and elevate it as a ground for legal reform (Fossa, 2021). The second is broader and relates to the relationships between the different domains at stake. Despite the important interactions between philosophical and legal analysis, whether RAI should gain something akin to an electronic personhood is only partially dependent on the moral status of such technologies and should thus be discussed in a proper legal perspective. While the legal and philosophical approaches find some points of convergence in the discussion on what properties would make RAI “moral agents,” they diverge whenever the focus is on whether, and if so how, attributing them legal entitlements would foster the ends of the legal system.

The distinction among different research questions forces us to be analytically clear and coherent. On the one hand, it teaches us against conflating the issue of whether robots may be bearer of rights and duties in general with that of their responsibility and accountability. On the other, it forces us to choose, among the various manifestation of “standing,” what exactly to address in the substantive part of the inquiry, precisely to avoid unpreceded claims, whose province and implications would be hard to tame.

Disentangling the two possible approaches according to which something may or should qualify as a subject is equally fundamental. First, before arguing for a solution based upon a specific approach, it is important to question whether the latter is accepted by the system under analysis—moral or, in our case, legal—and, if so, which role it may legitimately play. Secondly, arguing an identical conclusion in terms of policy recommendation bears radically different theoretical and practical consequences, depending on which of the two perspectives is adopted. If we say that robots should be held responsible because they are the “subjects”—and not a mere tool in the hands of a human—thence not only their liability may follow but also complex bundles of rights and obligations intended to protect their own interest. Even if the original question only concerns RAI’s standing as per their accountability, the solution would impact their overall legal status. The discussion on rights and duties on the one side, and liability on the other, would ultimately converge. Instead, if RAI are treated as juridical persons with the sole aim of segregating selected assets (shielding human beings from the legal and economic consequences of its operations, and eventually providing a diversified taxation scheme), then the overall legal—and ethical—implications radically differ (Bertolini, 2013; Pagallo, 2018a). The two stances must not be confused.

We will now address the question—are or should RAI qualify as subjects legally accountable for their own actions—following the various steps introduced above.

## Law, Morality, and the Grounds of Legal Subjectivity

Law and morality are two normative systems that control and regulate social behaviors, which may be framed as at least partly independent. In a legal system, positive and enforceable standards of conduct guide a community, preventing conflicts and incentivizing desirable behaviors, and offer second-order criteria for identifying, modifying, and enforcing said rules (Marmor and Sarch, 2019).^3^ On the contrary, morality comprises of those principles that society deems relevant for distinguishing between right and wrong (Gert and Gert, 2020), and offers a code of conduct valid irrespective of what is legally enacted. The ultimate relationship between the two domains constitutes one of the oldest and major questions in jurisprudential studies (Bentham, 1823; Austin et al., 1954; Dworkin, 1977; Dworkin, 1986; Wren et al., 1990; Coleman, 2003; Kelsen et al., 2005; Raz, 2009; Finnis, 2011; Hart, 2012; Hershovitz, 2015; Dickson, 2021). Positivist theories hold that legal normativity is autonomous and distinct from that of morality, and its validity is not dependent on its content (Green and Adams, 2019). Naturalist theories, on the contrary, argue that law and morality are interdependent and that to regulate social behaviors, the law must have moral content (Finnis, 2020).

Yet—even the most extreme of the naturalist theorists would concede—the moral relevance of a matter cannot be considered *per se* the source of legal normativity and deriving one from the other would be a serious mistake. This does not mean that moral considerations have no role in the legal domain, but rather that they must be contextualized within the space attributed to them by the legal system. If we want to discuss the legal status of RAI—the starting point needs to be the grounds upon which a given order qualifies entities, for the sake of regulation (Bryson et al., 2017). The question then becomes: how does a legal system “decides” how to qualify different entities? (Kurki, 2019).

Regulation—and legal reforms in particular—may be grounded in two different approaches (Bertolini, 2013).

According to the “ontological” or “essentialist” perspective, entities have a clear-cut legal qualification based on their inherent features, which in turn determines the applicable legal rules. Pursuant to such a narrative, we may need to adopt new rules, or change existing ones, when the object of the regulation (in this case, RAI applications) is so different from what we have been regulating so far (other, less advanced forms of technology), that a distinct legal qualification is due. In the current debate, such a stance claims the necessity to elaborate an alternative and potentially intermediate category between that of subjects and objects of law (Calo, 2015). The first notion encompasses those that within the legal system are attributed rights, the latter those entities upon which rights insist, and are exerted by the former. However, so defined, the duality of the alternative appears logically necessitated, to the point that an intermediate category would be altogether inconceivable and useless in a technical legal perspective. Indeed, either one entity is solely capable of being subject to someone’s rights—hence it’s an object –, or is able to possess rights. *Tertium non datur*. The circumstance that the law treats some entities such as corporations possessing rights for the sake of a given legal relation, while, in others, considers them as the objects upon which rights are exerted, simply means that the distinction between subject and object may be contingent upon different legally relevant circumstances, and does not lead to the existence of an intermediate category (Kurki, 2019).

If this is true from the ontological perspective, the functional one has quite a different approach. Indeed, the latter claims that legal frameworks shall be developed according to their adequacy in performing the functions attributed to them, as well as the broader consequences deriving therefrom. In this view, a particular legal qualification, and the rules applicable thereto, should be adopted based on the desirability of social, legal, and economic implications they bring about (Bertolini, 2013; Bertolini, 2014; Balkin, 2015a; Palmerini and Bertolini, 2016; Bryson et al., 2017).

Our legal systems commonly work on a combination of the two approaches: there are specific features that justify the qualification of an entity as a legal subject and, in addition, *ad hoc subjectivity* is sometimes granted for functional reasons. Regardless of whether these represent “legal fictions,” or mere expression of the legal system’s normative power to recognize rights and duties, what is important is that the two approaches may very well coexist.

The following paragraphs further elaborate on this point, disentangling and critically evaluating the various arguments underlying the call for “artificial personhood” under both the ontological and the functional perspectives.

## RAI as *Subjects*? The Ontological Perspective

Both the idea that we shall avoid the so-called “responsibility gap”—where humans are forced to compensate damages for which they have no or very limited control, and that machines shall behave as responsibly as possible, according to the principles elaborated through “machine ethics” (Wallach and Allen, 2009a; Anderson and Anderson, 2011) –, and that according to which RAI may have rights of their own, are often expressly grounded on the belief that the peculiar features displayed by advanced RAI (their asserted autonomy and ability to modify themselves) make them agents; more specifically, moral and possibly legal subjects, who should consequently be held responsible for their actions (Allen et al., 2005; Wallach and Allen, 2009b; Howard and Muntean, 2017).

However, the ontological claim according to which RAI’s essential qualities make them subjects, rather than mere objects, is far from being proved (Fossa, 2018).

This consideration, begs for a further question. Indeed, if we are to define what a robot can and cannot do by referring to the notions of subjectivity or personhood, agency, responsibility or liability, it is first necessary to understand what we mean by these concepts, which have complex and possibly indeterminate meanings.

As anticipated above, when discussing the challenges and opportunities brought about by RAI, both economic, legal, ethical, philosophical, and engineering considerations come into play, leading the debate to merge the methodological and analytical background of heterogeneous disciplines. Yet, economists, engineers, philosophers, and lawyers may use terms that have both a common, a-technical understanding and one which is peculiar of their own subject. Therefore, engineers or lawyers may speak of autonomy to denote different qualities than the ones that philosophers understand as associated with the said notion (Haselager, 2005). This constitutes a case of semantic ambiguity. Both the meaning of a concept and the conditions of its use depend on the context in which the latter is used, so that the transmission of a notion from one context to the other represents a process of “semantic extension,” which may lead to substantial confusion (Waldron, 1994; Endicott, 2000).

As highlighted by the studies on legal reasoning and linguistic indeterminacy (Waldron, 1994; Endicott, 2000), unclear and under-specified terminology may compromise the acceptability of the warranties used to back a specific argument, which in turn affects the correctness of the overall claim (Toulmin, 1964; Alexy, 1978).

### The Philosophical Notion(s) of Subjectivity and Agency

Trying to identify and condense the philosophical debate on what is a “subject” is a dauntingly difficult task and one which is not our intention to embark on. In essential terms, we may define a subject as an entity that relates with another entity that exists outside itself—the object— through a relationship which the subject enters by means of personal experience and/or consciousness (Thiel, 2011).

In continental philosophy, the discussion on “subjectivity” strongly relates to that of “agency” and “moral status”. In this section, we will consider the former, while § 4.3 will discuss the latter.

From a philosophical perspective, agents are subjects who can act—i.e., perform actions—while agency denotes the manifestation of such capacity (Schlosser, 2015). However, “actions are doings, but not every doing is an action” (Himma, 2009): according to the main variations of the “standard conception”, an event may be deemed as an action only if brought about intentionally (Anscombe, 1957; Davidson, 1963) thus not being the mere result of causal determinations among naturalistic events.

In turn, intentionality is often defined as “the determination of a specified end that implies the necessity of actions of a specified kind” (Gutman et al., 2012).

According to some authors, the kind of rationality required for intentional performance consists in being capable of rationally justifying one’s actions in reference to determined and determinable purposes, which, in turn, requires the deliberative and argumentative skills that only human beings possess, let alone because of their linguistic abilities. Under this view, only humans can perform actions, being able to reason and decide intentionally (Frankfurt, 1971; Taylor, 1977; Gutman et al., 2012).

Other theories set a lower threshold, describing intentionality as a mental state—such as belief, desire, will—that does not necessarily entail human-like rationality, and rather extends to the spontaneous initiation of actions that do not follow rationally justifiable desires (Ginet, 1990). Pursuant to this idea, “X is an agent if and only if X can instantiate intentional mental states capable of directly causing performance” (Himma, 2009).

However, this begs the question of how to detect mental states, whether they are non-physical subjective experiences or rather objective attitudes in the physical structure of the entity. Even if the very essence of mental states is difficult to grasp, some still read them as requiring a certain capacity of introspection, and thus of consciousness—but how to determine its existence, or set the relevant threshold required, is uncertain (Himma, 2009). Against this “hard problem”, some suggested to presuppose consciousness, unless proved otherwise, and treat an entity as having such capacity based on the performative equivalence of their doings with those of beings whose consciousness is not contested (Dennett, 1991; Frankish, 2016; Dennett, 2018).

In opposite terms, some authors have theorized a “minimal agency” which contests the need for “mental states” and qualifies as agent any unified entity that is distinguishable from its environment and that is doing something by itself according to certain goals. Pursuant to this view, very simple organisms can be said to have the intrinsic goal of continuing their existence, even if they lack the ability to rationally elaborate and justify their aims and actions (Barandiaran et al., 2009; Gunkel, 2018, pp 96-105).

The discussion of the qualification of RAI as agents is strongly debated, and it would fall beyond the scope of the paper (as well as the capacity of the authors) to solve it once and for all.

Nevertheless, from the above discussion, we can derive an important insight: the definition of agency constitutes a more basic notion than other compound concepts, such as those of rational, conscious, introspective, autonomous agency and the like (Himma, 2009). While it is possible to consider an agent as a “subject,” it is debatable that a mere agent—so loosely defined, without reference to rationality, consciousness, and intentionality—would meet the threshold relevant for legal consideration in an ontological perspective.^4^

As we will see in the following sections, this specification is of crucial importance also because, despite the variety of discourses which are made on the topic, the statement that RAI applications should qualify as agents—and thus be held morally and legally responsible—is based precisely on the (not always explicit) assumption that they are not mere agents, but rather *autonomous agents*.

Indeed, the idea of intentionality certainly goes towards (without necessarily overlapping) that of autonomy. Margaret Boden famously claimed that: “[a]n entity is autonomous when its behaviour-directing mechanisms may be shaped by the entity’s experiential history, are emergent in nature, and are reflectively modifiable by that entity”, deriving from this that “an individual’s autonomy is the greater, the more its behavior is directed by self-generated (and idiosyncratic) inner mechanisms, nicely responsive to the specific problem-situation, yet reflexively modifiable by wider concerns” (Boden, 1996). In similar terms, Gutman and colleagues define an autonomous entity as one whose actions are i) free, in the sense of resulting not from external coercion but rather from one’s own deliberation and ii) are means to achieve ends which are set by the subject himself (Gutman et al., 2012). Condition i) sets the standards that we have already discussed, namely, that an action is to be contrasted to a mere behavior, a deterministically caused event that was not brought about intentionally. What differentiates the notions of intentionality and autonomy is that the latter puts major importance on the origin of the goals for which the actions are performed. Defining an entity as an autonomous agent—instead of a mere agent—implies that the former has acted to obtain its own goals.

### The Legal Notion(s) of Subjectivity and Agency

As a social construct, the definition and attribution of legal personality is subject to historical and cultural changes. Indeed, twenty-first century developments—such as the raise of environmentalist and animalist concern, as well as artificial intelligence, and corporate personhood—compelled us to critically consider who, or what, is a “person” according to the law, and how our understanding of legal personhood came about (Kurki, 2019).

In the modern western legal tradition, the “orthodox view” (Kurki, 2019) sees legal subjectivity or personhood as the capacity to hold legal positions, such as rights and duties.^5^ Each person has said status from the moment of birth until their death, being banned forms of *capitis deminutio*, such as those related to slavery in ancient Rome or to political and racial prosecution of Jews in the Nazi regime.^6^ This means that the exclusion of legal personhood to certain categories of human beings is prohibited, although foreign national or stateless person may lack the capacity to hold some rights, with the exclusion of human rights, which belong to everyone because of their human being. In a specular way, embryos and fetuses are also granted specific safeguards, and may be attributed ad-hoc legal rights—particularly some personal rights (like that to health) and patrimonial (heirship)—despite not qualifying as “natural persons”.^7^

However, legal capacity is not an exclusive feature of human beings: non-human entities—such as corporations and associations—may be granted general legal capacity, thus being capable to bear those rights and duties which do not require the holder to be a human being (thus excluding, e.g., those arising from marriage). Organizations set up to undertake an activity may thus qualify as “persons” and treated as autonomous and separated from the natural persons owning and administering them—although in exceptional occasions the veil of asset partitioning can be lifted, making shareholders personally liable for the debts of the corporation (Kraakman et al., 2017, 5 ff).

Thus, in the legal dimension, being an agent equals having “legal capacity,” whereas a narrower version of this notion merely covers the “legal capacity to act”.

Indeed, despite possessing legal personhood, legal subjects may still lack the legal capacity to act, i.e., the ability to autonomously modify one’s rights and duties by performing legal acts. This constitutes a first fundamental definition of “agency” in legal terms.^8^

To be correctly understood, such notion shall be complemented with a taxonomy of legally relevant facts and acts, which—with some variations (e.g., in the legal, doctrinal, or jurisprudential formants—Sacco, 1991)—may be found in various jurisdictions belonging to the European continental legal tradition^9^.

Indeed, “facts” denote naturalistically caused events or human behaviors producing specific legal effects, where—if having human origin—it is immaterial whether they were brought about intentionally or not. On the contrary, “acts” constitute intentional actions which the law considers as the basis to produce given legal effects. Among the latter, we could further distinguish among: i) “mere acts”, where the action itself is intentional, but the legal effects are produced regardless of whether the author intended to bring about such legal consequences or not; ii) “juridical acts”, which produce their peculiar legal effects only if the action was performed intentionally as a means to achieve specific consequences; said otherwise, the production of legal effect is not a mere by-product of the action, by rather the reason why the latter was undertaken.

What has been said so far does not mean that the actions of those who lack the legal capacity to act have no legal effect, or that they do not have the power to perform legal actions at all. On the contrary, any entity—even non-human entities—may cause events, for which the law sets specific legal consequences, despite no legal capacity being required therefor. For a person to perform mere acts, it is necessary to have what is called “natural capacity”, i.e., having the ability to understand the meaning and consequences of one’s own actions, and to act accordingly. For example, if an underage child, having full intellectual capacity, causes physical damage to another person with fault or malice, she would still be liable for the wrong caused (even though, under certain conditions, her parents would be called to bear the economic consequences). On the contrary, full legal capacity is required for entering a valid contract or performing other juridical acts. If we assume that the same under-age person may be a real-estate owner and wanted to sell a property, despite having the legal capacity (as far as the ability to be entitled with property rights is concerned), she would lack the power to enter a legally valid contract, and need someone else acting on her behalf, namely an agent. This leads us to another point worthy of discussion.

Indeed, in a narrower sense, the term “agency” also refers to that institution, or rather set of norms, allowing and regulating the fiduciary relationship whereby a subject—the “agent”—is expressly or implicitly authorized to act on behalf of another subject—the “principal”—to create legal relations between the latter and third parties. Thus, an agent who acts within the scope of authority conferred by his or her principal—or so long as a third party in good faith may legitimately believe her to do so—binds the principal to the obligations she creates vis-a-vis third parties. However, for such effects to be produced, it is not necessary for the agent to have legal capacity, but only for the principal.

Against this background, the relevant question then becomes whether RAI could be “legal subjects” and, if so, whether they could only cause legal fact or also legal act. As for the first issue, it seems that the alternative is either recognizing the fully fledge status comparable to that of “natural persons,” if they are deemed to have essentially similar features to that of humans (and no functional reasons justifying not doing so!) or attribute them ad hoc legal personhood similarly to what we do with corporations. While the second option is, in technical terms, possible and compatible with the tools offered by the legal systems, the first one depends on our understanding of the relevant properties that would make a robot sufficiently like us, to justify its qualification as a legal subject (Kingwell, 2020; Osborne, 2020; Jowitt, 2021)—which we seek to identify throughout this paper.

For the moment, it is interesting to consider the second question addressed below, namely, whether RAI could perform legal acts. As for legal act *stricto sensu*, the question is again, whether their autonomous actions could qualify as “intentional” for the purpose of the legal system. Otherwise, it would constitute merely a legal fact. If, on the contrary, it could produce such an effect, then the behavior would qualify as a legal act and possibly a juridical act. However, from a legal perspective, this does not mean that robots would necessarily become fully-fleged subjects: their role may resemble that of the agent, who acts towards the end set by the principal, and thus produces effects within the legal sphere of the latter, being able to choose how to perform the intended task—including, for instance, concluding contracts. Indeed, the law allows the production of effects on another subject, who is held responsible for having identified the desired results, regardless of the level of autonomous agency displayed by the entity who performed the action. Just like a person may be bound to the legal effects produced in her legal sphere by the contract signed by a representative—an adult with full legal capacity, who has the maximum autonomy in determining the content of the agreement—, she may as well be bound by the effects produced by the action of a machine—certainly showing a lower degree of autonomy than the corresponding human agent—whose activity was initiated or requested by him, and who identified the need the system was to fulfil.

### RAI as *Accountable* Subjects? The Philosophical Notion(s) of Responsibility

According to the traditional philosophical discourse based on Aristotleian ethics (Aristotle, 1985), moral responsibility is the state which characterizes the subject whose actions are judged as worthy of praise or blame (Eshleman, 2016).

According to the perspective adopted, moral responsibility may be either merit-based—so that praise or blame would be an appropriate reaction toward the candidate only if s/he deserves such reactions—or consequence-based—so that moral judgment would be appropriate only when they are likely to have the desired effect in the agent’s actions and dispositions –. In this paper, we will take into consideration the merit-based approach, as the major reactions to morally reprehensible actions take the form of legal sanctions (broadly intended, i.e., considering different forms of liabilities) (Bobbio, 1969; Hart, 2012). The consequence-based approach to moral responsibility, on the contrary, shall thus be reframed as a peculiar form of the functional approach to the ascription of liability, which will be considered in the following section.

In this sense, one’s action may be a candidate for moral evaluation, only if she i) could exercise control over her actions and dispositions, and ii) was aware of what she was bringing about. These are generally referred to as the control and the epistemic conditions (Eshleman, 2016).

For the sake of this argument, we will leave aside the deterministic problems connected to one’s ability to control her actions and dispositions, and merely assume that i) agents have a certain degree of freedom of determination and ii) the practice of holding someone responsible needs no external justification in the face of determinism, since moral responsibility is based on social intrinsic reactive attitudes (Strawson, 1962).

That being said, it is necessary to ask whether a machine could meet the control condition. Again, this question must be addressed considering the peculiar form of “autonomy” that current RAI display. Indeed, they lack what is commonly referred to as “strong autonomy,” i.e., the ability to decide freely and coordinate one’s action towards a chosen end, and only have a “weak autonomy,” i.e., the capacity to decide, without external input or human supervision, between different possible ways of performing a given task or achieving a given goal. Even in a scenario where the machine learns from the environment, possibly adapting its functioning as a result of this interaction and learning, the machine cannot be said to be in control of its actions: even if it is free to determine the way in which to act, its choice is still determined by the need to interactively adjust its functioning to the environment and, on the basis of the available data, plan the most efficient way of performing its tasks. Given that the machine does not have control over the goals which it is programmed to achieve, since they are set by humans (most likely, the programmer), it cannot be deemed in control of the end itself (Gutman et al., 2012; Bertolini, 2013).

Likewise, artificial moral responsibility could not be recognized because it would still lack the epistemic condition. In the philosophical debate, the issue of awareness is separated by that of the possible deviancy of the causal chain initiated with one’s own actions, which, if anything, shall be traced to the definition of agency, not of moral responsibility (Schlosser, 2015). Awareness is rather to be understood as “the interpretive process wherein the individual recognizes that a moral problem exists in a situation or that a moral standard or principle is relevant to some set of circumstances” (Rest, 1986). One entity’s complete and unavoidable lack of moral awareness equals the impossibility of its moral consideration (Brożek and Janik, 2019).

As of now, machines lack cognitive skills (Searle, 1980; Searle, 1984; Koops et al., 2010; Gutman et al., 2012), and, it is unlikely that, at least in the near future, they will be capable of properly understanding the moral significance of their actions. Despite researchers’ attempt to ‘design artificial agents to act as if they [were] moral agents’ and make them sensible to the ‘values, ethics and legality of activities’ (Allen et al., 2000; Allen et al., 2005; Lanzarone and Gobbo, 2008), a series of problems arise: the first one lies in the very definition of the ethical principles to be encoded, upon which disagreement is likely to be found; the second one is related to ambiguities connected to the use of natural language, which may lead to gaps and incongruences between what the robot is told to do, and what the designer actually intended it to do—as it is everything but trivial to translate normative statements into strings of commands; the third one is rather connected to the peculiar functioning of ethical norms, as well as many legal norms, which do not apply once and for all, but may be subject to conflicts, exceptions and balancing, which require processes of prioritization and proportionality assessments, which are far from easy to be pre-defined in a way as to be hard-coded in the machine.

Said otherwise: machines can certainly perform actions which are, in abstract terms, worthy of reactive moral attitudes; however, since they cannot engage in moral considerations, they will not qualify as moral subjects, and thus may not be attributed moral responsibility (Himma, 2009 correctly notes that all the three capacity of moral agency—rationality, ability to know the difference between right and wrong, and the ability to apply correctly these rules to certain paradigm situation that constitute the meaning of the rule –, and indeed the very concept of agency, requires the agent’s consciousness).

In this sense, it is worth highlighting how the theories which accommodate artificial moral agents are often based on formal definitions and behavioristic tests that aim at proving that there is no qualitative difference between artificial and human agents. A famous example for this is the thesis offered by Floridi and Sanders, who claim that moral responsibility shall be equated to the ability to cause moral effects, which arises when an entity satisfies the formal criteria of interactivity, autonomy, and adaptability (Floridi and Sanders, 2004).

However, it has been recently demonstrated how such claims shall be read within the perspective of the machine ethics projects, and do not hold absolutely. The theoretical possibility of constructing a theory that is functional to the attribution moral agency to robots, assimilating robots and humans, does not mean that, in absolute terms, there is no significant difference between the two, nor that there is a pragmatic reason why artificial moral agency shall be constructed (Fossa, 2018).

Said theories may also be more radically challenged, for they deconstruct the notion of agency and responsibility, providing a more limited and alternative meaning to that generally accepted in the philosophical and legal discourse, yet failing to argue the reason why such an alternative proposal ought to be accepted. Said otherwise, why moral agency ought to be defined as the possibility to produce morally relevant consequences, irrespective of any identifiable intention and awareness,^10^ which are instead identified as a requirement by all moral and legal paradigms, is itself to be questioned. On the one hand, their philosophical admissibility is not self-evident. On the other hand, as per their practical implications, so conceived, they are useless. Holding a machine responsible that does not fear the sanction, deprives the legal norm of its primary purpose, namely that of inducing a desired behaviour on the side of the agent.

Ultimately, RAI applications do not share human’s autonomy and moral awareness necessary according to an absolute—i.e., non-instrumental or sector-specific—definition of moral agency, as the latter “cannot abstract from the very determination of ultimate ends and values, that is, of what strikes our conscience as worthy of respect and concretization” (Fossa, 2018).

### RAI as *Accountable* Subjects? The Legal Notion(s) of Liability

In legal terms, being liable means to be responsible or answerable for something at law. It rests on the idea that there are specific sources of obligations, which bind one subject to do something, denoted as the object of the obligation.

In criminal matters, liability arises because of a court decision, when the prosecutor demonstrates beyond reasonable doubt that the defendant’s conduct meets both the mental and the physical elements required for the offence to be punished under criminal law, and consists in fines and imprisonment, as well as other non-custodial punishments. Under western legal tradition, criminal liability has a sanctioning, as well as a re-educative aim.^11^

Civil liability rules determine who is supposed to bear the negative economic consequences arising from an accident, and under which conditions. Here, liability means “the law determining when the victim of an accident is entitled to recover losses from the injurer” (Shavell, 2007a). Typically, the party is held liable, and thence bound to compensate, that is deemed to have caused the accident, and therefore is responsible for it. Liability is established after a trial, where the claimant, who sued the wrongdoer, must prove the existence of the specific constitutive elements that ground the liability affirmed. Under English civil law, for example, to hold a person liable for negligence, the claimant needs to prove that the defendant had a duty, that she breached it, and that such breach caused an injury, resulting in recoverable damages; for instance, because the harm is not too remote a consequence of the breach (Van Gerven et al., 2000).^12^

Civil liability rules pursue three distinct functions, namely: i) ex-ante deterrence, since they aim at making the agent refrain from the harmful behavior, given that she will have to internalize the negative consequences caused; ii) ex-post compensation of the victim, as they force the person responsible for the damage to make good for the loss suffered; (iii) and ex post punishment, since the compensatory award also constitute a sanction, making sure that the infringer does not get away with the illicit behavior.

Many different theories have been elaborated to justify civil liability, as well as to shape its rules within a legal system according to specific ideologies; most of them are related to a different notion of justice. According to a retributive account of justice, the blameworthy deserve to suffer, because of the socially reprehensible character of their conduct, and liability rules shall be framed to serve as sanctions (Walen and Winter 2016). Theories of corrective justice, instead, understand tort law as a system of second-order duties (Coleman, 2003), setting obligations to make good the wrong caused by the breach of first-order duties; under this view, liability rules shall rather be elaborated and interpreted to assure that the victim is put, as much as possible, in the position she would be, had the damage not occurred. Thus, for a loss to be wrongful and worthy of being compensated, it needs to derive not from morally reprehensible conduct, but rather from a damaging violation of the victim’s right.^13^

In Law&Economics (L&E) theories, liability rules constitute economic incentives, leading agents to adopt economically efficient behaviors, which increase the overall social benefit. In this sense, paying damage is almost equal to buying the right to obtain the benefit associated with the wrong (Calabresi, 1970; Calabresi and Melamed, 1972; Shavell, 2007b; Polinsky and Shavell, 2007).

Nowadays, legal systems do not commit to only one theory of tort and justice, but rather to a combination of the three: the same normative framework will feature different models of liability rules, displaying a variety of imputation criteria (causation/remoteness, subjective elements), which in turn reflect the peculiar rationales underlying the attribution of liability.

Many tort law systems—such as the Italian one^14^—have a general rule prescribing liability for damages caused by reprehensible behaviors based on fault. This solution is moved by all the different goals defined above: not only ex-post compensation and sanction but also ex-ante deterrence, since fault-based liability incentivizes agents to adopt the standard of care necessary to avoid harmful behaviors, and thus the negative economic consequences deriving from the duty to compensate.

Sometimes, however, the defendant is held liable in tort even though she did nothing blameworthy, merely because of the particular position that the she holds towards the cause of the damage: i.e., the person who has a duty to watch over some other entity—such as the keeper, owner or user of a dangerous thing, the keeper or user of an animal—, or the person who benefits from having or using a thing, or running a specific activity.^15^ The basic idea underlying the ascription of liability is that who has the economic or otherwise benefits associated with possessing or running a dangerous thing or activity, should also make sure that no damages are caused, and pay whenever this happens. This model is often associated to a strict or semi-strict liability, depending on whether the defendant may exclude his duty to compensate—i.e., by demonstrating that he took all the necessary measures to prevent the harm to occur, or by demonstrating that the latter was caused by an act of God—. The stricter the liability, the more compensation-oriented, instead of deterrence- and punishment-oriented the rationale.

Further down this line, sometimes liability is ascribed to the person who is best positioned to manage and internalize the risk, preventing its occurrence and minimizing its consequences, as well as to compensate the victim once an accident occurs. Such a model is particularly common in L&E literature (Polinsky and Shavell, 2007).

A peculiar version of this model is the so-called Risk Management Approach (henceforth RMA), which is grounded on the idea that liability should not be attributed based on considerations of fault—defined as the deviation from the desired conduct—typical of most tort law systems, but rather on the party that is best positioned to i) minimize risks and ii) acquire insurance. It moves from the basic consideration that—despite liability rules may well work as incentives or disincentives towards specific behaviors–they may not ensure sufficient and efficient incentives towards a desirable ex-ante conduct, be it a safety investment—such as in the case of producers’ liability—or a diligent conduct—such as the driver’s in the case of road circulation—, and that end is best attained through the adoption of the detailed ex-ante applicable regulation, such as safety regulation. According to this view, liability rules should thus be freed from the burden of incentivizing the agents towards desired conducts, and rather be shaped to ensure the maximum and most efficient compensation to the victim. In extreme cases, this could also be designed as to avoid the difficulties and burdens connected to traditional judicial adjudication, and rather be based on no-fault compensatory funds (Bertolini, 2016).

## The Functional Perspective

In the previous analysis, we have clarified that for an entity to be deemed an agent, it shall be able to instantiate intentional mental states capable of directly causing performance and that for it to qualify as a moral agent, it shall display what is usually referred to as “strong autonomy,” i.e., the ability to decide freely and coordinate one’s action towards a chosen end, as well as the moral awareness needed for understanding the moral significance of one’s actions.

In doing so, we have also explained why current RAI, conceived to complete a specific task identified by their user, shall not qualify neither as agents, absent the consciousness required for them to have intentional mental states, nor as moral agents, given that, at this stage, they have no capacity to engage in moral judgments, and lack a “strong autonomy,” because they can determine how to reach the goals they are programmed to achieve, but said goals are defined by an external agent—most likely, the designer, producer or programmer—. The only moral agents involved in the functioning of the RAI application remain the humans behind it, who are responsible for its goals, its model of functioning, as well as for the very choice to grant to it a certain degree of autonomy in determining how to perform intended tasks (Putman, 1964; Bertolini, 2013; Nyholm, 2018; Dignum, 2020).

Having excluded any ontological reason why robots shall be deemed autonomous agents, thus moral and legal subjects, they shall be qualified as products: “artefacts crafted by human design and labor, for the purpose of serving identifiable human needs” (Bryson and Kime, 2011; Bertolini, 2013; Fossa, 2018). Therefore, should a robot cause any damage, ordinary product liability rules would apply. Since the latter rests on the idea the producer shall be responsible because, and as long as, he is in full control of the features and actions of the products (Bertolini, 2013), the proclaimed “responsibility gap” (Matthias, 2004; Koops et al., 2010; Calo, 2015) is then only apparent. Contract law typically allows a full-fledged autonomous and conscious human being to act—when so legitimized either by the law or by the free choice of the party—in the name and interest of another human being, immediately modifying his legal sphere (e.g., agency). Similarly, tort law allows one party to be called in to compensate the damage caused by another subject under his supervision that only at times displays limited capacity and awareness (e.g., underage child), and in other cases is instead as autonomous as the very party obliged to pay damages (e.g., an employee). In both cases, the legal system copes with a much higher degree of autonomy—that displayed by the autonomous human agents—by imputing the legal and economic consequences of their actions to another, entirely different human being, who has a very limited—much more limited than that possessed over a machine of any sort—control over their actions.

Regardless of the complexity of its functioning, as far as the RAI application performs the tasks it was designed for, it is still under the control of the producer or the programmer: even in the case of machine-learning technologies—such as neural-based systems and genetic algorithms—the unpredictability of the learning behavior does not create any actual lack of control, but rather requires the training and the associated evolution to be included in the development phase so that the product reaches the market only when it is supposed to have learnt or perfected the skill to function safely. Should such threshold be impossible to reach, so that the machine seems not to be able to develop in a predictable way, the moral and legal responsibility for the damage caused still lies on the producer/programmer, who has a duty not to put unsafe products into the market.

What has been said so far against the alleged responsibility gap served to prove that there are no compulsory ontological reasons why ordinary product liability rules shall not apply to advanced RAI. However, it could still be the case that changes to the extant paradigm shall be made, to address the regulation of new technologies, in a way that fosters technological innovation while being respectful of and driven by the respect of European values and principles (European Commission, 2018). Social and policy considerations, as well as constitutional law, may suggest the adoption of different liability models, favoring the development of applications that are particularly valuable for society, such as prostheses or devices intended to help the otherwise disabled in their everyday tasks (Bertolini, 2015).

Likewise, current liability rules may be rethought or reformed, to better pursue the goals that they are meant to achieve (Koops et al., 2010; Bertolini, 2013; Lior, 2019; Kiršienė et al., 2021). Indeed, the Product Liability Directive—which constitutes the European framework on the issue—has recently been evaluated to assess whether it is still adequate for regulating contemporary advanced technological products. Some critical elements have been identified (Expert Group on Liability and New Technologies, 2019; Bertolini and Episcopo, 2021): the uncertainty as per the qualification of software as a product, the undesired implications deriving from the development risk defense, the cost and difficulty of exactly ascertaining the existence of a defect—in particular in design –, as well as of a causal nexus between the fact and the damage. The latter burdens the claimant substantially, discouraging litigation. Also, when advanced robotics is considered, tight human-machine interaction causes different bodies of law to overlap. Indeed, if a single task is handled together by the human agent and by a machine, when an accident occurs it might be due to the fault of the former or a defect (or malfunctioning) of the latter. Apportioning liability among the two—human agent or manufacturer—might therefore require complex factual ascertainment and articulate legal analysis. For this purpose, different approaches—such as the abovementioned Risk Management Approach—have been elaborated, which suggest modifying current product liability rules to better address the new challenges brought about by technological innovation (Bertolini, 2013; Bertolini, 2014).

### The Benefits of a Functionally Attributed Electronic Personhood

Even if RAI cannot qualify as autonomous beings and, thus, there is no ontological reason why they should be considered as subjects at law, it does not mean that they may not qualify as such, because of the discretional choice of the legislator, so long as the latter is well grounded on sound policy analysis.

Indeed, the constitutive independence among the notion of personhood, agency, and responsibility in the moral and legal domains is such that functional reasons could very well justify a dissociation between the different states. For example, ad hoc legal personhood could be awarded to robots, exactly as it is granted to corporations. However, to justify this choice specific end needs to be identified, and a comparative judgment on the pros and cons of this alternative, as well as other tools, shall be considered. For example, it may be useful to attribute it to robotic applications, such as software agents to be used on capital markets which would then be registered, as to identify the limits of its allowed tasks and functions, and eventually the (physical or legal) person it is representing.

With respect to liability issues, the recognition of legal personhood would mainly serve as a liability capping method; yet it would neither necessarily change the person bearing the costs of its functioning nor the cases when compensation is awarded. Unless the robot could earn revenues from its operation, its capital would have to be provided by a human, or a corporation, standing behind it, thus not necessarily shifting the burden from the party that would bear it pursuant to existing product liability rules. Such a result could also be achieved through insurance mechanisms or with a simple damages cap. Should the robot be allowed to earn a fee for its performance, this would only constitute a tax on the user, producing an overall risk-spreading effect which could be effectively achieved otherwise, for instance through the adoption of a no-fault scheme funded by the product’s users in various fashions (Bertolini, 2013; Expert Group on Liability and New Technologies, 2019). Which of the different alternatives is preferable is still a matter of correctly specifying particular circumstances, among which are the size of the market for the given application and the existence of evident failures which could be designed around through ad hoc regulation; much less would depend on the machine being weakly autonomous or even able to learn.

In this sense, we do not share the radical exclusion of legal personhood which, for instance, Bryson and colleagues make, based on the asserted undesirability of the consequences associated with such solution (Bryson et al., 2017). Indeed, the authors claim that the recognition of a legal personality—although technically possible—would be “morally unnecessary and legally troublesome”: in their view, legal personhood may have some emotional or economic appeal, but difficulties in holding “electronic persons” accountable when they violate the rights of others outweigh the highly precarious moral interests that RAI’s legal personhood might protect. On the contrary, we argue that, although that may be the case in some circumstances—so that the humans behind them should be held responsible under the above-described risk-management approach—, other cases may well justify the recognition of such legal status, provided that said legal personality is narrowly and functionally defined (against a one-size-fits-all approach; see, e.g., Dahiyat, 2021).

## Conclusion

The major issue faced when discussing the possibility to attribute i) subjectivity, ii) agency, and iii) responsibility to RAI is the lack of clarity in identifying the nature of the argument that may be either ontological or functional. The two paradigms lead, in fact, to divergent considerations, and should thence always be kept profoundly distinct.

On the contrary, conclusions reached in the current debate often appear ambiguous because they tend to mix the two separate perspectives. Such lack of clarity is further advanced by the constant—and otherwise beneficial—exchange between lawyers and philosophers, who utilize those apparently similar notions with very different purposes and conceptual frameworks of reference.

While in some philosophical domains the lack of intentionality might appear insufficient an argument to exclude agency, and thence responsibility, such (de- and re-)constructions may not be transposed in the legal domain. There, intentionality serves an unavoidable purpose, that of ensuring the possibility of deterrence through regulation. The norm, by threat of a sanction, induces the desired behaviour only in those entities that are aware of their own existence, possess individual preferences, and are capable of freely coordinating their actions to achieve them. The lack of any of such elements excludes the very utility of attributing responsibility and eventually sanctioning the transgressor.

At the same time, the legal system is well structured to deal with the need to impose legal effects produced by the behaviour of a subject onto another one, despite the former being fully capable of determining himself autonomously, so long as the latter identifies the ends to be achieved.

If we look at the specificities of the legal system, it is then objectively observable that there are no ontological grounds to determine the attribution of subjectivity, rights, duties, and obligations to machines. Nothing in the way the machine is designed, functions, or is justifies the legitimacy of such attributions, rather excludes them.

However, a functional analysis may lead to different conclusions so long as adequate purely legal arguments could be identified. That may only be achieved with respect to i) specific classes of applications, ii) when a technical—in a legal perspective—need is identified, that is best pursued through the attribution of rights and obligations to the machine itself, rather than the humans behind it. In such a sense, the attribution of agency, subjectivity or responsibility would not follow the acknowledgment of a special nature of the RAI, but of a legal need for—separately or jointly—a) simplification of legal relations, b) traceability, registration and transparency of the entity and of those possessing interests in it and in its operation, c) segregation of assets and limitations of responsibility.

Based on those considerations, conclusions such as those reached by the EU parliament in its recent proposal on the regulation of civil liability for AI—whereby since “[…] all physical or virtual activities, devices or processes that are driven by AI systems […] are nearly always the result of someone building, deploying or interfering with the systems […] it is not necessary to give legal personality to AI-systems” (European Parliament, 2020 introduction n° 7)—appear excessively broad and thence unjustified. Said otherwise, the mere circumstance that all legal relations revolving around corporations could be described and regulated through bundles of contracts, does not justify *per se* the exclusion of the utility of legal personhood. The reason such a conclusion is flawed in a technical legal perspective has to do with the technology-neutral approach that proposal attempts to maintain, presenting a uniform regulation for applications and use cases that are extremely different one from the other, and that today would be addressed by entirely different branches of the legal ordering (such as capital markets, traffic accidents, medical or professional malpractice, to name a few).

It is indeed certain that the cases in favor of the direct attribution of liability to a RAI application need to be individually justified, yet that debate belongs entirely to the technical-legal domain and has no bearing nor implications on the acknowledgment of the machine as an entity deserving moral standing and the attribution of individual rights.

## Data Availability

The original contributions presented in the study are included in the article/Supplementary Material, further inquiries can be directed to the corresponding author.

## Footnotes

1 In the generalist press, see, respectively: https://www.reuters.com/technology/google-self-driving-spinoff-waymo-begins-testing-with-public-san-francisco-2021-08-24/, https://www.theverge.com/2020/12/9/22165597/cruise-driverless-test-san-francisco-self-driving-level-4; https://www.nationalgeographic.com/photography/article/sophia-robot-artificial-intelligence-science; https://www.businessinsider.com/meet-the-first-robot-citizen-sophia-animatronic-humanoid-2017-10, https://www.forbes.com/sites/zarastone/2017/11/07/everything-you-need-to-know-about-sophia-the-worlds-first-robot-citizen/?sh=475456c46fa1 (all articles last accessed on 15 December 2021). For a recent review of the debate, see Schröder, W.M. (2021). “Robots and Rights: Reviewing Recent Positions in Legal Philosophy and Ethics,” in *Robotics, AI, and Humanity: Science, Ethics, and Policy,* eds. J. Von Braun, M. S. Archer, G.M. Reichberg and M. Sánchez Sorondo (Cham: Springer International Publishing), 191–203.

2 Research topic description, available at https://www.frontiersin.org/research-topics/17908/should-robots-have-standing-the-moral-and-legal-status-of-social-robots (last accessed 15 December 2021).

3 A simple yet effective overview of this view can be found under the entry “Legal Positivism,” offered in the Oxford Dictionary of Law (John Law (ed), Oxford: Oxford University Press (2018)): “An approach to law that rejects natural law and contends that the law as laid down (*positum*) should be kept separate—for the purpose of study and analysis—from the law as it ought morally to be. In other words, a clear distinction must be drawn between “ought” (that which is morally desirable) and “is” (that which actually exists). The theory is associated especially with the thought of Jeremy Bentham (1748–1832), John Austin (1790–1859), H. L. A. Hart (1907–1992), and Hans Kelsen (1881–1973), who differ from one another in important respects but generally adhere to the above separability thesis. In addition, legal positivists normally adopt the so-called social fact thesis (that legal validity is a function of pedigree or related social facts) and the conventionality thesis (that social facts giving rise to legal validity are authoritative by virtue of social convention)”.

4 Indeed, functional considerations might lead to different conclusions, but there it is not the notion of agency in its philosophical dimension that matters, see §5.

5 In recent times, the concept of legal personality has been challenged by external pressures: the limitation of “natural personhood” to human beings is allegedly harder and harder to justify, but the legalist alternative of “everything goes” is condemned as unworkable and counterproductive. Against these considerations, the very notion of legal personality is undergoing a new phase of scrutiny. Some have gone so far as to contest the correctness of the “orthodox view,” suggesting that legal personality should be seen not as a gradual property, where some essential elements of a broader “bundle of personality incidents” are attached to an entity Kurki, V.a.J. (2019). *A theory of legal personhood*. Oxford, United Kingdom: Oxford University Press. These suggestions are certainly worthy of careful considerations. Yet, the critique to the notion of legal personality seems unnecessary, as it is the rejection of the binary alternative between legal subjectivity and lack thereof. While a proper discussion of the matter would fall outside the scope of this paper, it is here important to recall that two important contributions made by said renewed conception could be incorporated within the traditional—and commonly accepted—understanding of legal personality. First, the idea of legal subjectivity as a boundless of incidents can be usefully incorporated in the understating of legal persons as “entities capable of holding legal positions,” in the sense that it helps clarifying the various configurations that legal personhood may have. Indeed, only humans are considered has having a fully-fledged legal personality, whereas other entities may well be recognized as subjects whenever attributed specific rights and duties, without automatically acquiring the capacity to hold other forms of entitlements. The distinction that Kurki makes between legal subjects and entities that merely qualify as rights or duties holder is arguably better framed as acknowledging different degrees or extensions of legal capacity.

6 In Italy, for example, natural persons acquire legal capacity with birth (art. One of the Italian Civil Code), and no one can be deprived of it for political reasons (art. 22 Italian Constitution). References on this matter may only be minimal Falzea, A (1989). “voce « Capacità (teoria gen.)»,” in: *Enciclopedia del diritto* (Milano: Giuffrè)., 8 ff.

7 On the legal status of embryos and fetuses, Jost, T.S. (2002). Rights of Embryo and Foetus in Private Law. *American Journal of Comparative Law* 50., Seymour, J (2002). The legal status of the fetus: an international review. *J Law Med* 10**,** 28–40.

8 According to our previous example (the Italian legal system), one subject acquires the capacity to act when he or she become of age—turns 18 years old—(art. Two Italian Civil Code) and can be limited or revoked by the courts, for example through interdiction, i.e., by depriving the person of the right to handle his or her own affairs because of mental incapacity (art. 414 ff. Italian Civil Code). See Stanzione, P (1988). “voce «Capacità I diritto privato»,”, in: *Enciclopedia giuridica* (Bologna-Roma: Zanichelli-Foro it.).

9 Indeed, variations on these distinctions exist between different legal systems. The tripartite structure is typical of German, law, which differentiate between juridical facts, juridical acts, and legal transaction. On the contrary, French law expressly differentiate only between juridical acts—legal transactions—and juridical facts, but the latter are thought to encompass both what we here identify a juridical facts *stricto sensu* and juridical acts *stricto sensu*, and indeed attaches different legal consequence to each category. Italian law, instead, distinguishes between “fatti giuridici” and “atti giuridici,” but legal scholarship follows the German model, and it predominantly (although not unanimously) acknowledges the category of “negozi giuridici” (i.e., legal transactions) as opposed to that of “atti giuridici in senso stretto” (i.e., other legal acts). For a synthetic but effective reconstruction of this issue, see Sirena, P (2020). *Introduction to Private Law*. Il Mulino.

10 The notion so conceived also denies the minimal prerequisite of *suitas*, Padovani, T (2002). *Diritto Penale*. Milano: Giuffrè. 111-112, whereby the absolute lack of any intention prevents the very possibility of assessing any (criminal) responsibility of the agent. Indeed, the latter notion builds upon and is deeply rooted in the philosophical debate in this subject matter.

11 See art. 27 Italian Constitution: “Le pene (…) devono tendere alla rieducazione del condannato”.

12 For leading cases on the tort of negligence and on compensatory damages arising therefrom, see Donoghue v. Stevenson [1932] AC 532, 580; *Nettleship v Weston* [1971] 2 QB 691; *Smith v Leech Brain and Co.* [1962] 2 QB 405; *The Wagon Mound No.2* [1967] 1 AC 617 Privy Council.

13 Under some version of this theory—developed to object other forms of liability, as developed by the school of law and economics—the principle of corrective justice that justifies the link which tort law creates between the victim and injurer, since it takes the injurer to have the duty to repair the wrongful losses that he causes, and neatly considers compensation as the primary function of liability, against that of inducing efficient behaviour.

14 Art. 2043 Italian Civil Code: «*Risarcimento per fatto illecito*. Qualunque fatto doloso o colposo, che cagiona ad altri un danno ingiusto, obbliga colui che ha commesso il fatto a risarcire il danno».

15 See, e.g., Wagner, G (2015). “Comparative Tort Law,” in *Comparative Tort Law,* eds. M. Reimann and R. Zimmermann (Oxford: Oxford University Press).
